# Leber’s hereditary optic neuropathy and multiple sclerosis: overlap between mitochondrial disease and neuroinflammation

**DOI:** 10.3389/fneur.2025.1538358

**Published:** 2025-02-18

**Authors:** Golbarg Rahimi, Mackenzie Silverman, Maeve Lucas, Lilia Kazerooni, Mariam M. Yousuf, Saba Jafarpour, Jonathan D. Santoro

**Affiliations:** ^1^Keck School of Medicine of the University of Southern California, Los Angeles, CA, United States; ^2^Division of Neurology, Department of Pediatrics, Children’s Hospital Los Angeles, Los Angeles, CA, United States; ^3^Department of Neurology, Keck School of Medicine of the University of Southern California, Los Angeles, CA, United States

**Keywords:** mitochondrial dysfunction, Leber’s hereditary optic neuropathy, multiple sclerosis, neuroimmunology, neuropathology, Harding’s syndrome

## Abstract

Although Multiple sclerosis (MS) and Leber hereditary optic neuropathy (LHON) have distinct pathophysiological mechanisms, they are both neurodegenerative conditions that involve mitochondrial dysfunction. MS is an autoimmune disease that is characterized by demyelination and neuroinflammation; and LHON is a mitochondrial disorder predominantly affecting the optic nerves, resulting in severe vision loss. Recent studies have highlighted the coexistence of these two conditions, particularly in females, suggesting that mitochondrial variants in LHON may predispose individuals to develop MS or affect its progression. Similar to MS, LHON-MS presents with visual impairment, neurological deficits, white matter lesions, and brain atrophy, which further supports a shared underlying pathophysiology. While MS is not inherently a mitochondrial disorder, its neuroinflammatory processes can lead to mitochondrial dysfunction. Reciprocally, mitochondrial impairment may be exacerbated in LHON-MS. Therefore, the role of mitochondrial dysfunction in these diseases is central, with impaired mitochondrial function contributing to cellular damage and neuroinflammation. This review explores the intersections of MS and LHON, emphasizing the need for further research to better understand mitochondrial dysfunction in these disorders.

## 1 Introduction

Multiple sclerosis (MS) is a chronic autoimmune disease that leads to inflammation, demyelination, and neurodegeneration in the central nervous system (CNS) (1). The primary pathology underlying this disease is axonal demyelination. Lesion location and burden contribute to the variable clinical presentation and disease severity seen in this population. The disease is more common in women than in men, with a ratio of about 3:1, and in individuals of Northern European descent (2). In fact, MS is one of the most common autoimmune neurologic conditions worldwide with about 2.9 million individuals diagnosed (3). While optic neuritis (ON) can commonly be seen in MS, co-morbid mitochondrial eye disease has also been described in this population.

Leber hereditary optic neuropathy (LHON) is a mitochondrial disorder characterized by bilateral central vision loss due to degeneration of retinal ganglion cells and the optic nerves. It is most commonly seen in young males (4–6). Several mitochondrial DNA variants which disrupt complex I of the respiratory chain have been associated with the disease (6–9). Interestingly, these variants have incomplete penetrance with only about 50% of males and 10% of females with these mutations ultimately developing the condition. This suggests additional genetic, epigenetic, and environmental factors may be involved (6, 10). In addition to the complex genetics at play, mutations may also be synergistic in some cases, producing specific phenotypes (11). As there are no definitive or curative treatments for LHON, the condition is considered neurodegenerative and progressive in most cases, although emerging interventions such as gene therapy are on the horizon (12, 13).

Previous reports have described cases of LHON and MS co-occurrence. This overlap is predominantly seen in female patients (14). It has been proposed that harboring LHON variants is a risk factor for developing MS (15). It is possible that mitochondrial DNA variants in persons with MS (PwMS) could potentially contribute to unique clinical subgroups, emphasizing the need for further investigation into the overlapping pathophysiology of LHON and MS (16). This narrative review will explore the complex interactions between mitochondrial disease and neuroinflammation to enhance our understanding of the shared mechanisms underlying these disorders.

## 2 Clinical and radiographic overlap in LHON and MS

The overlap between LHON and MS has gained attention in recent days, such that LHON-MS has been given a distinct name, Harding’s Disease (17). This association was initially reported by Lees et al. in 1964, who observed that LHON and MS could co-occur in the same individual (18). Harding et al. further expanded on this observation, documenting 11 cases in which LHON and MS symptoms coexisted, supporting the idea that LHON mutations might predispose individuals to neuroinflammation and associated conditions such as MS (17).

Recent studies support this association. An extensive review involving 55 LHON families and 40 patients with confirmed MS highlighted that primary LHON mutations are associated with an increased risk of developing MS (15). Notably, all three primary LHON mutations identified in European and North American populations (m.11778A > G, m.3460A > G, and m.14484 T > C) have been associated with symptoms similar to those of MS, including vision problems, motor function issues, and cognitive difficulties, suggesting a genetic predisposition (15). This association is particularly evident in females despite LHON predominantly affecting men, indicating that MS may be triggered in women with LHON when certain environmental factors are present (14, 18).

LHON-MS shares certain features with MS including age of onset, a female predilection, and a predominance of the relapsing–remitting MS phenotype (71.1%). However, it differs significantly in that 96% of LHON-MS patients experience visual involvement with only 10% reporting ocular pain and 72.1% lacking visual recovery, resulting in 50% of patients registering as legally blind. In contrast, only 50% of patients with isolated MS have visual involvement with 85 to 95% recovering to better than 6/9 visual acuity (19–21). These features may be important when differentiating these disease processes as painless ON and lack of visual improvement with treatment would be atypical for isolated neuroinflammatory disorders (Table 1). This suggests that LHON-MS has a distinct clinical phenotype that may reflect a unique mechanistic interaction between LHON and MS.

**Table 1:** Clinical differences between LHON, ON, LHON-MS, NMOSD, and typical MS (6, 17, 19).

	Typical MS	NMOSD	Optic neuritis (ON)	LHON	LHON-MS
Sex ratio (Female:Male)	2:1	4:1	3:1	1:4	2:1
Peak age of onset	20–40 years	30–40 years	25–40 years	20–35 years	20–35 years
Ocular pain	Yes with ON	50%	>90%	Rare, <10%	10%
Visual recovery	Common, >85% recover	Limited acutely	Common, >90% in MOGAD	Minimal, progressive	~25% acutely although typically progressive
Acute response to steroids	Yes	Yes although less profound, often requiring plasma exchange	Yes, rarely requires escalation to plasma exchange	Limited	~50%
Progression to blindness or visual disability	Rare	Uncommon but present in severe cases	Rare	Common	~50% of cases
Cortical lesions on MRI	Necessary for diagnosis	Uncommon	Rare, may indicate another diagnosis or association with MOG antibodies	Not anticipated	Common, necessary for diagnosis

Leber’s hereditary optic neuropathy (LHON), Leber’s hereditary optic neuropathy and multiple sclerosis (LHON-MS), myelin oligodendrocyte glycoprotein (MOG), myelin oligodendrocyte glycoprotein associated disease (MOGAD), multiple sclerosis (MS), neuromyelitis optica spectrum disorder (NMOSD), optic neuritis (ON).

**Figure 1:**
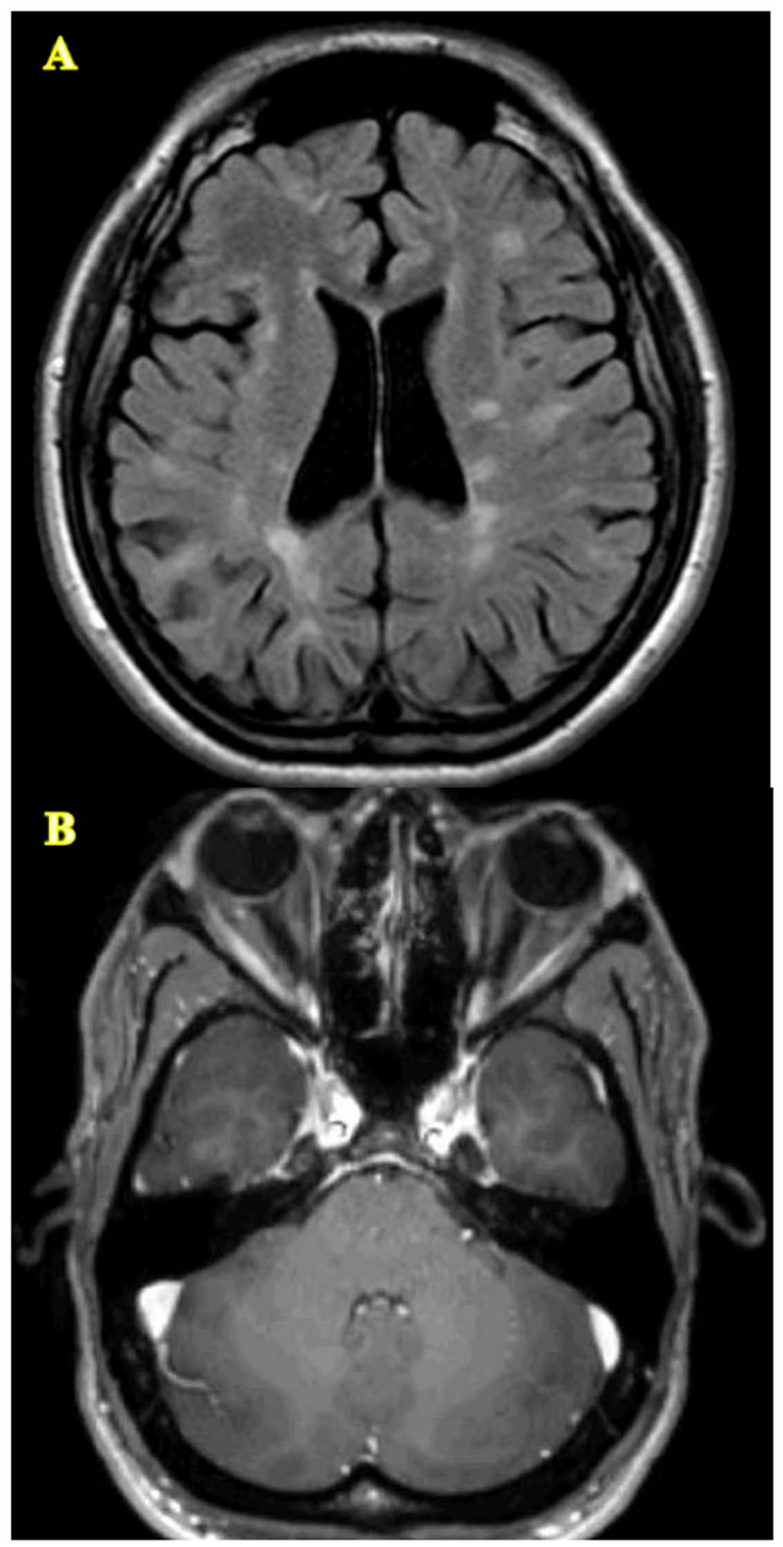
Lesions of the CNS characteristic of LHON-MS. **(A)** Diffuse T2/FLAIR signal abnormalities throughout the white matter meeting McDonald’s 2017 criteria. **(B)** Atrophy of the optic nerves.

Neuroimaging studies show that the imaging characteristics of LHON-MS closely resemble those of MS (Figure 1) (14, 17). Per McDonald’s 2017 criteria, neuroimaging abnormalities in MS typically occur in multiple areas of the CNS, showing patterns of indistinguishable white matter lesions and brain atrophy, which have also been reported in individuals with LHON-MS (14, 22). As compared to LHON alone, neuroimaging in patients with LHON-MS demonstrates more extensive white matter abnormalities and optic nerve damage (23).

## 3 LHON and mitochondrial dysfunction

LHON is a mitochondrial disorder that leads to severe visual impairment or blindness due to the degeneration of retinal ganglion cells (RGCs). The disease is mainly caused by point mutations in mitochondrial DNA (mtDNA) affecting the complex I subunits of the electron transport chain, which leads to less effective cellular energy production and higher susceptibility to cell death (24). The typical clinical presentation includes subacute bilateral visual loss, central scotoma, dyschromatopsia, and eventual optic disk atrophy, with early stages marked by pseudoedema and microangiopathy (6, 25).

**Figure 2:**
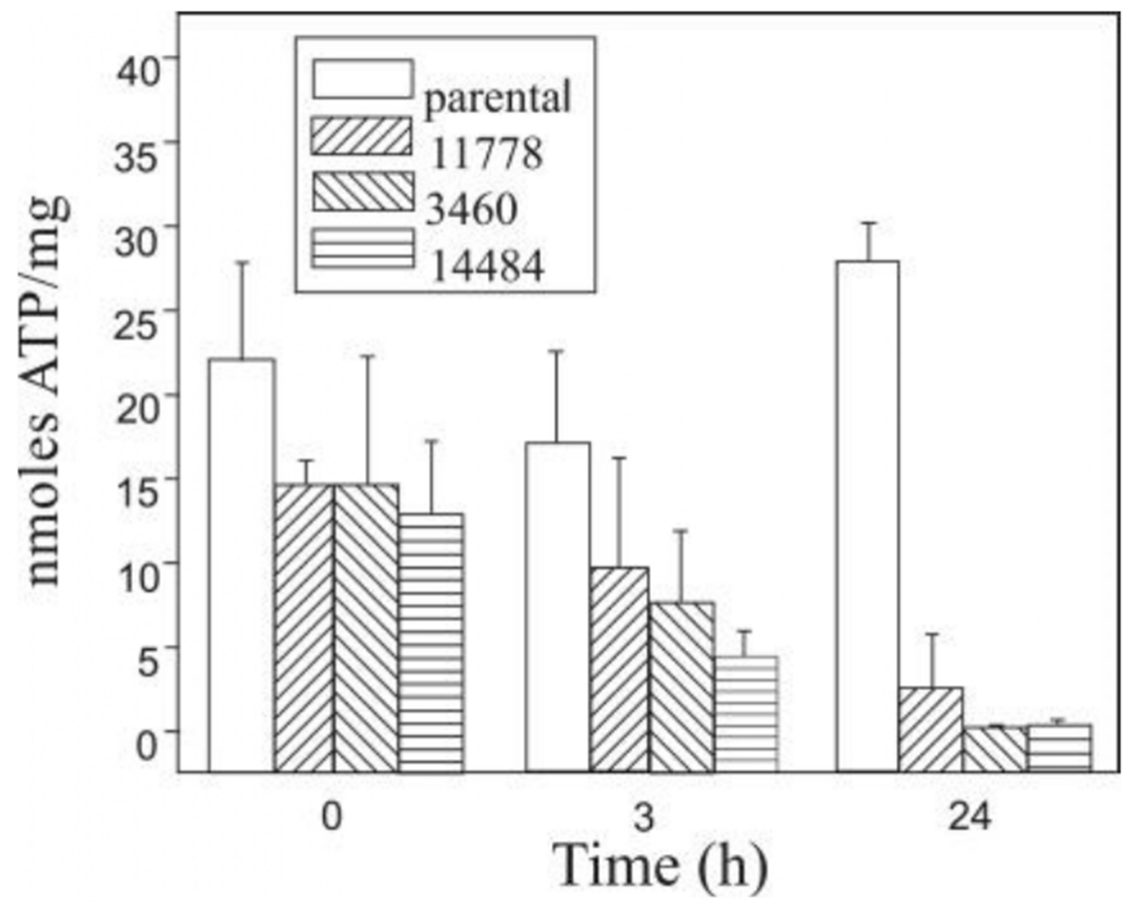
ATP content in 142B.TK- cybrid cells, harboring the three most common primary LHON mutations, and in the parental cell line were determined in triplicate after various incubation periods in galactose-medium (27).

The role of mitochondrial dysfunction in the pathogenesis of LHON is well established. Ghelli et al. demonstrated that osteosarcoma-derived cytoplasmic hybrids (cybrids) with common LHON mutations (11,778/ND4, 3,460/ND1, and 14,484/ND6) undergo apoptotic cell death when under metabolic stress induced by galactose, which leads cells to rely on mitochondrial respiration for ATP production (26). This study showed that LHON cybrids exhibit signs of apoptosis such as chromatin condensation, nuclear DNA laddering, and increased cytochrome c release into the cytosol, with mutations 3,460/ND1 and 14,484/ND6 leading to greater apoptotic susceptibility compared to 11,778/ND4 (Figure 2) (24).

In addition to mitochondrial dysfunction, the regulation of superoxide production in RGCs compared to brain and neuroblastoma cells has been explored. Research by Levin found that RGCs produce superoxide at lower rates than brain mitochondria with tighter regulation, potentially preventing aberrant apoptosis signaling (25). Disruption of this balance by LHON-related mtDNA mutations could lead to increased superoxide levels, contributing to RGC death and optic neuropathy (25, 27).

The complexity of diagnosing LHON is in part due to its similarity to other conditions such as neuromyelitis optica spectrum disorders (NMOSD), isolated ON, and MS. Accurate diagnosis relies on high clinical suspicion and comprehensive diagnostic resources (Table 1), with definitive diagnosis achieved through identifying specific mtDNA mutations (Table 2) (28). Recent findings have also identified new autosomal recessive mutations in patients with LHON-like symptoms, broadening the spectrum of the disease (29).

**Table 2:** Mitochondrial DNA variants identified in individuals with LHON (13).

Genes	Nucleotide position	AA change	Phenotype
MT-ND1	m.3316G > A	A4T	LHON/NIDDM
	m.3376G > A	E24K	LHON/MELAS
	m.3394 T > C	Y30H	LHON/NIDDM
	m.3460G > A	A52T	LHON
	m.3496G > T	A64S	LHON
	m.3497C > T	A64V	LHON
	m.3635G > A	S110N	LHON
	m.3700G > A	A112T	LHON
	m.3733G > A	E143K	LHON
	m.4025C > T	T240M	LHON
	m.4136A > G	Y277C	LHON
	m.4160 T > C	L286P	LHON
	m.4171C > A	L289M	LHON
	m.4216 T > C	Y304H	LHON/Insulin resistance
MT-CO1	m.6261G > A	A120T	LHON/Prostrate Cancer
	m.7444 G > A	Ter-K	LHON/SNH/DEAF
	m.7623\u00B0C > T	T13I	LHON
MT-CO2	m.7868C > T	L95F	LHON
MT-ND2	m.4640C > A	I57M	LHON
	m.4917A > G	N150D	LHON/AMD/Insulin resistance/NRTI-PN
	m.5244G > A	G259S	LHON
MT-ND3	m.10237 T > C	I60T	LHON
MT-ND4	m.11253 T > C	I165T	LHON
	m.11696G > A	V312I	LHON + Spastic Dystonia
	m.11778G > A	R340H	LHON and LHON/MS
	m.11874C > A	T372N	LHON
MT-ND4L	m.10543A > G	H25R	LHON
	m.10591 T > G	F41C	LHON
	m.10663 T > C	V65A	LHON
MT-ND5	m.12782 T > G	I149S	LHON
	m.12811 T > C	Y159H	LHON
	m.12848C > T	A171V	LHON
	m.13045A > C	M237L	LHON/MELAS/LS
	m.13051G > A	G239S	LHON
	m.13379A > C	H348P	LHON
	m.13528A > G	T398A	LHON-Like
	m.13637A > G	Q434R	LHON
	m.13708G > A	A458T	LHON/MS risk
	m.13730G > A	G465E	LHON
MT-ND6	m.14568C > T	G36S	LHON
	m.14279G > A	S132L	LHON
	m.14459G > A	A72V	LHON + Spastic Dystonia
	m.14482C > G	M64I	LHON
	m.14484 T > C	M64V	LHON
	m.14495A > G	L60S	LHON
	m.14498C > T	Y59C	LHON
	m.14596A > T	I26M	LHON
	m.14325 T > C	N117D	LHON
	m.14729G > A	S132L	LHON
MT-CYB	m.14831G > A	A29T	LHON
	m.14841A > G	N32S	LHON
	m.15257G > A	D171N	LHON
	m.15674 T > C	S310P	LHON
	m.15773G > A	V343M	LHON
	m.15812G > A	V356M	LHON
MT-CO3	m.9438G > A	G78S	LHON
	m.9738G > T	A178S	LHON
	m.9804G > A	A200T	LHON
MT-ATP6	m.8836A > G	M104V	LHON
	m.9016A > G	I164V	LHON
	m.9101 T > C	I192T	LHON
	m.9139G > A	A205T	LHON

Inflammation is initiated through the activation of pattern recognition receptors (PRRs) present in both immune and non-immune cells, which respond to microbial elements and endogenous signals known as damage-associated molecular patterns (DAMPs) (30). Under normal physiological conditions, DAMPs, including ATP and specific proteins, remain sequestered within cellular compartments and are unable to activate PRRs. However, cellular stress or death can alter membrane permeability, thereby facilitating the release of DAMPs and the subsequent initiation of inflammatory responses (30). Mitochondria play a pivotal role in this process for several reasons: they possess evolutionary similarities to bacteria indicating a potential interaction with PRRs; their dual membrane structure allows for the regulation of mitochondrial DAMP release; and they are crucial in mediating various forms of regulated cell death that promote DAMP redistribution and PRR activation (31–33). Thus, mitochondria serve as integral components in the regulation of inflammation, linking cellular stress responses to immune activation and contributing to the maintenance of homeostasis (34, 35). As such, it remains possible that in individuals with LHON-MS, mitochondrial dysfunction may be triggering a secondary immune phenomenon.

## 4 Possible mitochondrial dysfunction in MS

Mitochondria play a key role in the generation of cellular energy and various metabolic functions in neurons and oligodendrocytes (36). Mitochondria in oligodendrocytes play a vital role in providing the necessary energy for myelin synthesis, which is critical for proper neuronal function (36). They also contribute to controlling lactate availability, which helps with axonal function. Furthermore, mitochondria regulate calcium levels and have the ability to induce apoptosis when there is an excess of calcium, especially during periods of ischemia (36). A recent study used fluorescent markers in mitochondria to gain understanding of how they behave dynamically in the myelin sheath (36). These markers revealed that mitochondria are spread out in the myelin sheath, with the greatest concentration located in the cytoplasmic ridges beside the axon (36, 37). This distribution indicates that mitochondria play a specific role in maintaining the integrity of myelin and the health of axons (38). Electron microscopy has shown that mitochondria in oligodendrocytes have a smaller cristae surface area than those in neurons, suggesting a decreased capacity for ATP production (36). The results emphasize the significance of mitochondrial function in sustaining the energy demands of oligodendrocytes and supporting cognitive functions while also providing a potential neuroprotective therapeutic avenue for future study (39, 40).

Mitochondrial dysfunction is increasingly recognized as a critical factor in the pathophysiology of MS (41, 42). The depletion of myelin results in heightened energy requirements for neurons, due to the extensive participation of the axonal membrane in depolarization, resulting in elevated ATP usage (43). The heightened need may surpass the mitochondria’s capability to generate ATP, leading to compromised neuronal function. Mitochondria play a key role in producing ATP through oxidative phosphorylation (44). In MS, their impairment results in lower ATP production and increased oxidative stress (45). Research has shown that there is a higher amount of mitochondria and enzyme activity, specifically complex IV, in MS lesions than in normal-appearing white matter (46). Additionally, there is increased expression of mitochondrial stress proteins such as mtHSP70 in these regions. The neuroinflammatory environment characteristic of MS is also associated with increased production of reactive oxygen species (ROS), which are partly generated by dysfunctional mitochondria (47). This oxidative stress exacerbates inflammation and contributes to axonal damage and degeneration (48). These observations suggest that mitochondrial stress and oxidative damage are significant contributors to tissue damage and disease progression in MS (46). These pathological processes seem to yield a destructive potentiation cascade wherein further tissue damage also further impairs mitochondrial activity which has been directly associated with clinical disease progression (49). Moreover, mitochondrial inhibitors like rotenone have been shown to impair oligodendrocyte differentiation, further linking mitochondrial dysfunction to demyelination in MS (50).

These studies provide evidence that individuals with genetic disorders predisposing them to mitochondrial dysfunction may face an increased risk of developing MS or experiencing more severe disease manifestations. As inflammation has a detrimental impact on mitochondrial function, those with preexisting mitochondrial impairments could be more susceptible to the cellular damage seen in inflammatory processes such as MS and therefore have more severe disease presentation and rate of progression. While MS is not inherently a mitochondrial disorder, its neuroinflammatory processes can lead to mitochondrial dysfunction, a situation that may differ in LHON-MS, in which mitochondrial dysfunction is likely occurring in a bidirectional manner. These findings point to the critical role of mitochondrial health in MS severity and progression and highlight the potential of therapeutic targets aimed at mitigating mitochondrial dysfunction and oxidative damage (51, 52). Further studies are required to better characterize the impact of underlying mitochondrial dysfunction on clinical presentation and outcomes in patients with MS.

## 5 Therapeutic advancement in LHON

Recent treatment advancements for LHON have been targeted at treating mitochondrial dysfunction, which is fundamental to the disease’s pathology due to the impaired mitochondrial function in RGCs (53–55). Antioxidants such as Idebenone, a synthetic analog of coenzyme Q10, aim to enhance mitochondrial bioenergetics and mitigate oxidative stress (54). While initial studies suggested potential benefits in visual acuity (56), larger clinical trials such as the RHODOS study have produced mixed results (57). These findings highlight the complexity of mitochondrial involvement in LHON and elucidate the need for a deeper understanding of how mitochondrial dysfunction contributes to visual impairment.

Gene therapy has emerged as a promising approach, utilizing strategies such as allotopic expression to relocate mitochondrial genes to the nucleus which facilitates proper protein synthesis within mitochondria (58, 59). Early clinical trials targeting the ND4 mutation have shown some improvements in vision, suggesting that restoring mitochondrial function can yield positive outcomes, although with variable responses among patients (60). Additionally, emerging nutritional interventions such as the ketogenic diet aim to strengthen mitochondrial bioenergetics. Mitochondrial replacement therapy (MRT) also holds promise for preventing the transmission of LHON mutations by combining healthy mitochondrial DNA with nuclear DNA from affected individuals, although ethical considerations and technical challenges remain significant (61, 62). Given the potential curative role of these treatments and the ability to affect multiple areas of the body simultaneously, significant research efforts are likely in this area in the next few years (63, 64). Recent studies have also hypothesized that mitochondrial transplantation, which involves transferring healthy mitochondria into damaged cells, may be an effective treatment for MS (64).

## 6 Treatment overlap in LHON-MS

Disease-modifying agents for MS have never been tested for effectiveness in long-term disability reduction in LHON, as the established pathology of MS is likely distinct from LHON. Idebenone and Mitoxantrone have shown some benefit in LHON. Idebenone is considered safe and has led to visual improvements in some LHON cases (19, 57). Mitoxantrone has also shown some benefit but carries significant risks, making it less favorable compared to safer alternatives (65). On the other hand, LHON-MS may overlap significantly from both a clinical and radiographic standpoint with MS alone. Given the high likelihood of severe visual disability in LHON-MS patients, early intervention with disease-modifying therapies is recommended. Additionally, recent studies suggest that 4-aminopyridine may improve visual evoked potentials in certain MS patients, making it a potential candidate for further research in the LHON-MS population (66).

## 7 Future directions and research gaps

Despite emerging evidence linking mitochondrial dysfunction to both MS and LHON, significant gaps remain in understanding the specific roles of mitochondria in these conditions. Key areas where research is lacking include the precise molecular mechanisms by which mitochondrial dysfunction contributes to neuronal damage in MS and the interplay of genetic and environmental factors that may influence these processes. Furthermore, the extent to which mitochondrial mutations in LHON could predispose individuals to MS-like symptoms needs more investigation, particularly in diverse populations beyond those previously studied. Future studies should prioritize longitudinal investigations into mitochondrial dysfunction, assessing how these abnormalities evolve over time in MS and LHON patients.

## 8 Conclusion

The overlap of MS and LHON shines light on a complex relationship in which mitochondrial dysfunction plays a central role. The co-occurrence of these two conditions suggests that LHON mutations may predispose individuals to MS and possibly contribute to more severe clinical manifestations in patients. Both diseases exhibit neuroinflammation, visual impairment, and white matter lesions, highlighting the need for further research into how mitochondrial dysfunction influences disease progression. Understanding the mechanisms by which LHON mutations impact MS development remains an important area for future research, as it could lead to mitochondrial targeted interventions. As our understanding of mitochondrial involvement in these diseases grows, we will become closer to developing more effective, tailored therapies for patients suffering from both MS and LHON.
